# The BAF45D Protein Is Preferentially Expressed in Adult Neurogenic Zones and in Neurons and May Be Required for Retinoid Acid Induced PAX6 Expression

**DOI:** 10.3389/fnana.2017.00094

**Published:** 2017-11-06

**Authors:** Chao Liu, Ruyu Sun, Jian Huang, Dijuan Zhang, Dake Huang, Weiqin Qi, Shenghua Wang, Fenfen Xie, Yuxian Shen, Cailiang Shen

**Affiliations:** ^1^School of Basic Medical Sciences, Anhui Medical University, Hefei, China; ^2^Department of Histology and Embryology, Anhui Medical University, Hefei, China; ^3^Institute of Stem Cell and Tissue Engineering, Anhui Medical University, Hefei, China; ^4^Department of Spine Surgery, The First Affiliated Hospital of Anhui Medical University, Hefei, China

**Keywords:** BAF45D, adult neural stem cell, subgranular zone, subventricular zone, ependymal cells, PAX6

## Abstract

Adult neurogenesis is important for the development of regenerative therapies for human diseases of the central nervous system (CNS) through the recruitment of adult neural stem cells (NSCs). NSCs are characterized by the capacity to generate neurons, astrocytes, and oligodendrocytes. To identify key factors involved in manipulating the adult NSC neurogenic fate thus has crucial implications for the clinical application. Here, we report that BAF45D is expressed in the subgranular zone (SGZ) of the dentate gyrus, the subventricular zone (SVZ) of the lateral ventricle, and the central canal (CC) of the adult spinal cord. Coexpression of BAF45D with glial fibrillary acidic protein (GFAP), a radial glial like cell marker protein, was identified in the SGZ, the SVZ and the adult spinal cord CC. Quantitative analysis data indicate that BAF45D is preferentially expressed in the neurogenic zone of the LV and the neurons of the adult CNS. Furthermore, during the neuroectoderm differentiation of H9 cells, BAF45D is required for the expression of PAX6, a neuroectoderm determinant that is also known to regulate the self-renewal and neuronal fate specification of adult neural stem/progenitor cells. Together, our results may shed new light on the expression of BAF45D in the adult neurogenic zones and the contribution of BAF45D to early neural development.

## Introduction

Adult neurogenesis is important for driving structural plasticity of the brain through addition and integration of new neurons from adult neural stem cells (NSCs; Sailor et al., 2017), which can selfrenew and generate neurons, astrocytes, and oligodendrocytes (Gage, 2000; Ma et al., 2009). Aberrant modulation of the differentiation potential of NSCs residing in the central canal of the spinal cord contributes to cell loss and neural circuitry dysfunction following spinal cord injury (Sabelstrom et al., 2014). Disruption of the adult neurogenesis contributes to neurodegenerative, psychiatric, and cognitive diseases of central nervous system (CNS; Apple et al., 2017; Horgusluoglu et al., 2017). Therefore, it is crucial to develop regenerative therapies through employing the adult NSCs. Classically, adult NSCs can be identified in two discrete brain regions: the subgranular zone (SGZ) of hippocampal dentate gyrus (DG) and the subventricular zone (SVZ), which lines the lateral wall of the lateral ventricle (LV; Fuentealba et al., 2012; Apple et al., 2017; Horgusluoglu et al., 2017). In the hippocampal DG, the SGZ is located at the interface of the granule cell layer (GCL) and the hilus (Hil) and contains NSCs, which reside in a layer about three nuclei wide, including the basal cell band of GCL and two nuclei wide zone into the Hil (Balu and Lucki, 2009). Actually, there are different types of cells in the SGZ and the SVZ (Varela-Nallar and Inestrosa, 2013; Bonaguidi et al., 2016). Among the cells, the GFAP-expressing cells are believed to function as NSCs and are vital for the adult neurogenesis (Doetsch et al., 1999; Chojnacki et al., 2009; Mandyam, 2013; Varela-Nallar and Inestrosa, 2013; Bonaguidi et al., 2016). To identify key factors involved in manipulating the NSCs in the adult brain has important implications for the therapy of CNS diseases (Liu and Song, 2016; Ren et al., 2017). However, uncertainty about the identity of the adult NSCs remains.

Brg1/Brm-associated factor (BAF) chromatin remodeling complex arranges a switch in subunit composition between the neural subtype-specific BAF complexes to determine neural development and neural differentiation (Ronan et al., 2013; Narayanan and Tuoc, 2014). BAF45D, also known as DPF2, belongs to BAF45 family proteins, subunits of the BAF complexes, which include BAF45A, BAF45B, BAF45C, and BAF45D (Lessard et al., 2007). It is known that BAF45D mRNA is present in the developing cerebral cortex of mouse embryos at embryonic day 14 (E14; Gabig et al., 1998). In adult mouse brain, the presence of BAF45D in the hippocampus has also been addressed (Gabig et al., 1998). It is already known that BAF complex interacts with PAX6, a neurogenic fate determinant, and determines neurogenesis of adult NSCs (Ninkovic et al., 2013; Gotz et al., 2016). PAX6 is also a human neuroectoderm determinant (Zhang et al., 2010). Neuroectoderm is the primordium of human nervous system (Gammill and Bronner-Fraser, 2003). Moreover, PAX6 also contributes to brain structure and function in human adults (Yogarajah et al., 2016). Thus, in current study, we want to explore if BAF45D is expressed in adult nervous system and plays a role in PAX6 expression.

Here our data suggest that BAF45D is expressed in the SGZ of the adult DG, the SVZ of the adult LV and the spinal cord central canal. Coexpression of BAF45D and GFAP was identified in some of the cells in the SGZ, the ependymal cells of the SVZ, dorsal third ventricle (D3V) and the spinal cord central canal. Moreover, BAF45D is preferentially expressed in the neurons of the adult CNS. Further analysis revealed that retinoid acid (RA) downregulates OCT4 and upregulates PAX6, a neurogenic fate determinant, together with an upregulation of BAF45D during the neuroectoderm differentiation of H9 cells but fails to downregulate OCT4 and upregulates PAX6 upon knockdown of BAF45D. Besides, BAF45D siRNA does not affect significantly the expression of GATA6, an endoderm marker protein. These data may shed new light on the expression of BAF45D in adult neurogenic zones and function of BAF45D in early neural development.

## Experimental procedures

### Animals

Animals were acquired according to previously described (Duan et al., 2013). The adult mice (10–12 weeks old, weight 25–30 g) of C57/BL6 were obtained from the Experimental Animal Centre of Anhui Province.

### Tissue preparation

The preparation of adult animal tissues was performed as previously described (Duan et al., 2013; Lacroix et al., 2014). Briefly, the adult mice were anesthetized and sacrificed. Then the tissues of the brains and the spinal cords were dissected and post-fixed overnight in 4% paraformaldehyde at 4°C. Serial sections of 5 μm thickness were cut using a Leica microtome and mounted on CITOGLAS adhesion microscope slides.

### Cell cultures

A human embryonic stem cell line, H9 cells, was cultured as previously described (Liu et al., 2015). Briefly, H9 cells were maintained on Matrigel (BD Bioscience, Bedford, MA) in mTeSR medium (Stem Cell Technologies, Vancouver, BC, Canada) supplemented with 50 units per milliliter penicillin and 50 μg per milliliter streptomycin under 5% CO2 in a humidified incubator. The medium was changed daily and the cells were passaged at a ratio of 1:4.

### H9 cell neuroectoderm differentiation

H9 cells were infected transiently by lentiviruses containing negative control (NC) siRNA and BAF45D siRNA #25540, and were subjected to all-trans retinoic acid (RA, Sigma) induced neuroectoderm differentiation according to our previous protocol (Liu et al., 2015). Briefly, undifferentiated H9 cells were cultured in mTeSR supplemented with 10 mM Y-27632 at 37°C for 1 h. Then the cells were digested using accutase (Millipore) and washed with mTeSR. The cells were then centrifugated at 500 rpm/min and resuspended in 1 mL mTeSR. Then the cell suspension was incubated with 10 μL of concentrated GFP-expressing lentivirus containing DPF2 siRNAs (LV-DPF2 siRNA #25,540; Genechem, Shanghai, China) at 37°C for 1 h. Finally, the infected cells were supplemented with 10 mM Y-27,632 and replated on Matrigel-coated 6-well plates. The medium were change with mTeSR daily. When the lentivirus-infected H9 cells grew to 60–70% confluence, the cells were seeded in 6-well plates pre-coated with Matrigel at a ratio of 1:2 and maintained in mTeSR medium. The next day (d1), cells were exposure by all-trans retinoic acid at 10 μM in mTeSR medium. For a 3-day induction, the culture medium was refreshed with mTeSR medium containing 10 μM every day. At d4, the H9 cells were lysed and the lysates were subjected to IB.

### Immunohistochemistry (IH) assay

IH assay was performed as previously described (Duan et al., 2013; Lacroix et al., 2014). The sections were blocked with 3% goat serum and then incubated with mouse anti-PAX6 antibody (1:200, Millipore, Temecula, CA, USA) and rabbit anti-BAF45D (1:100, Proteintech, Chicago, IL, USA) antibodies overnight at 4°C. A NIKON Eclipse 80i fluorescent microscope and a NIKON Eclipse Ti-S inverted fluorescence microscope were used for visualization.

### Immunofluorescence (IF) assay

IF assay for tissue samples was performed according to a previous protocol (Gao and Chen, 2013). The samples were incubated with rabbit anti-BAF45D (1:100, Proteintech, Chicago, IL, USA), mouse anti-GFAP (1:100, Proteintech, Chicago, IL, USA), mouse anti-NEUN (1:100, Millipore, Belecula, CA, USA) and mouse anti-beta-III-tubulin (1:200, Millipore, Belecula, CA, USA) overnight at 4°C. After washed with PBS, the samples were incubated with Alexa Flour-488 anti-mouse (1:500) and Alexa Fluor-594 anti-rabbit (1:500) antibodies. The nuclei were counterstained with 4, 6-diamidino-2-phenylindole (DAPI). A NIKON Eclipse 80i fluorescence microscope and a NIKON Eclipse Ti-S inverted fluorescence microscope were used for visualization. In some cases, a Leica DMI6000CS confocal microscope was used for the visualization. More descriptions of the antibodies used for IF were shown in Table S1.

### Immunoblotting (IB) assay

The lysates of fresh tissues and the H9-derived cells were subjected to IB assay according to the previous protocol (Liu et al., 2012; Tripathi and Mishra, 2012). Then the proteins were detected using indicated antibodies. Antibodies used for IB are as follows: mouse anti-GFAP (1:500, Proteintech, Chicago, IL, USA), mouse anti-NEUN (1:500, Millipore, Belecula, CA, USA), rabbit anti-BAF45D (1:500, Proteintech, Chicago, IL, USA), rabbit anti-GATA6 (1:100, Abcam, New Territories, HK), mouse anti-OCT4 (1:1000, Santa Cruz Biotechnology, Santa Cruz, CA, USA), rabbit anti-PAX6 (1:500, Millipore, Belecula, CA, USA) and rabbit anti-GAPDH (1:2000, Proteintech, Chicago, IL, USA) antibodies. More descriptions of the antibodies used for IB were shown in Table S1.

### Ethics approval

All animal experiments were approved by the Anhui Medical University Experimental Animal Ethics Committee. The NIH Guide for the care and use of laboratory animals (National Institutes of Health Publications, No. 80-23, revised 1978) was followed for the acquisition and care of animals.

### Statistics

The statistical analysis was performed using SPSS17.0 software. Data for comparison of two groups were implemented by independent sample *T*-test and/or one-way ANOVA. For the proportion data belong to two groups of independent samples, we performed non-parametric statistics using Mann–Whitney *U*-test. The data are presented as the mean ± standard deviation. Statistical significance was accepted at *p* < 0.05.

## Results

### BAF45D is expressed in the SGZ of adult mouse hippocampal DG

**Figure 1:**
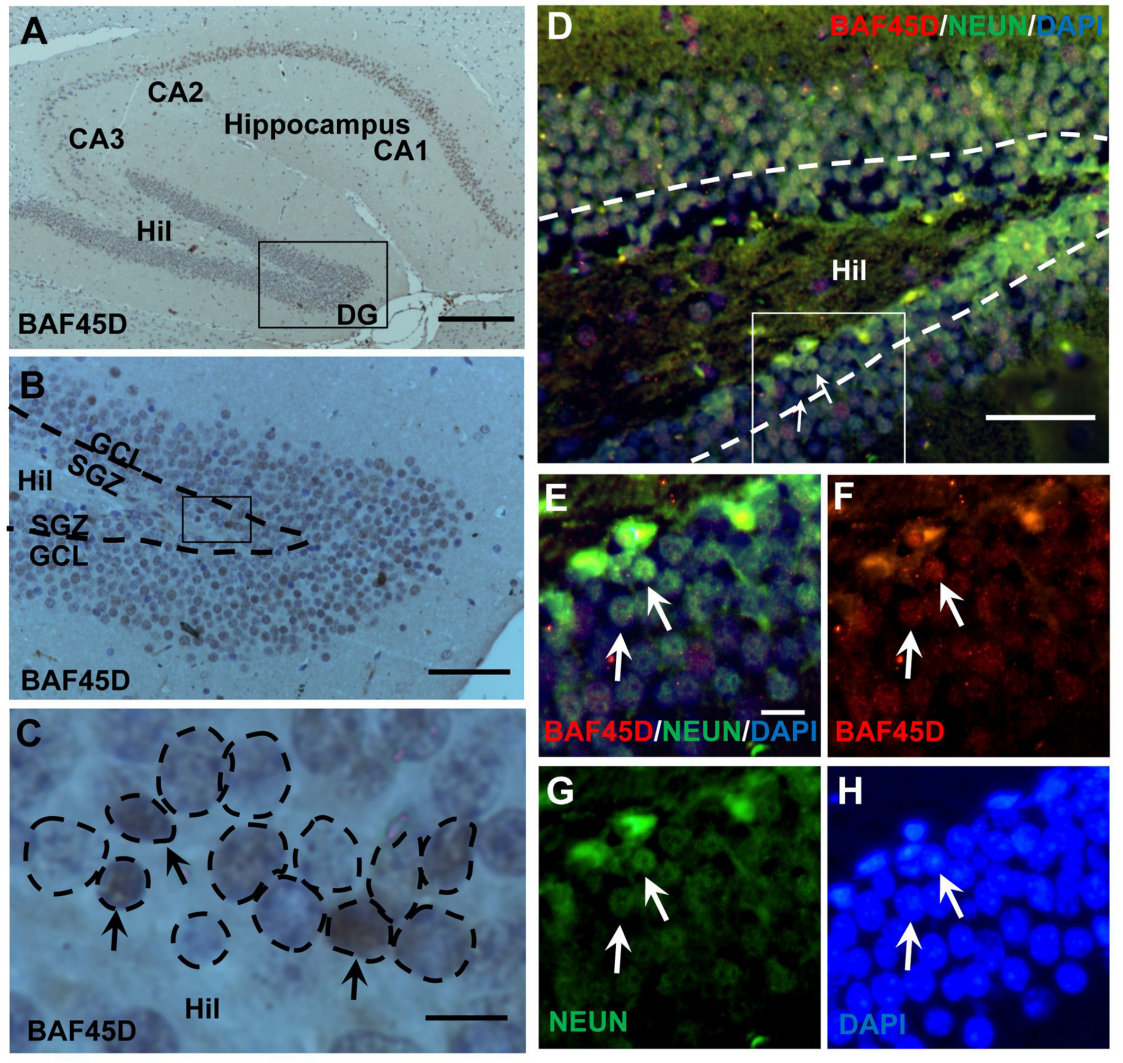
Expression of BAF45D in the adult hippocampal DG. **(A–C)** The sagittal sections (lateral 1.08 mm according to Paxinos and Franklin, 2001.) of the adult mouse brain were subjected to IH assay using anti-BAF45D antibodies. The BAF45D-immunopositive signals in the CA1, CA2, CA3, and DG regions of the hippocampus were shown **(A)**. Hil, hilus of the DG. Panel **(B)** is a higher magnification of the inlet in **(A)**. Panel **(C)** is a higher magnification of the inlet in **(B)**. The DG is characterized by the SGZ and the granule cell layer (GCL) **(B)**. The nuclear architecture of the SGZ is shown. The arrows indicate the BAF45D-immunopositive cells and the dashed circles indicate the various types of the nuclei **(C)**. **(D–G)** The DG section was subjected to IF assay using the indicated antibodies. Panel **(E)** is a higher magnification of the inlet in **(D)**. **(F–H)** are the indicated different signal channels that merged in **(E)**, respectively. Bar = 200 μm **(A)**, 50 μm **(B,D)**, and 10 μm **(C,E)**, respectively. The nuclei (blue) were counterstained by DAPI.

The SGZ of the hippocampal DG is a classical adult NSC niche. We performed HE staining and IH assay using the sections of the adult mouse brain cut in a sagittal plane, in which the CA1, CA2, CA3, and DG regions are shown (Figures S1A–C). Within the DG, both the SGZ and the GCL are also shown (Figure S1B). According to the IH assay data, as compared to the PBS control (Figures S1D–F), PAX6-immunopositive signals, although weak, were detected in the SGZ (Figures S1G–I). BAF45D-immunopositive signals were detected in the cells of the CA1, CA2, CA3, and the DG regions (Figure 1A). BAF45D is expressed mainly in the nuclei of the SGZ and GCL cells of the DG (Figure 1B). The 2–3 layers of the nuclei next to the hilus are shown (Figure 1C, dashed circles). We next performed IF assay using anti-NEUN and anti-BAF45D antibodies. The data indicates that BAF45D and NEUN, a mature neuron marker, are coexpressed in most of the DG cells (Figures 1D–H, arrows).

These results suggest that BAF45D is a nuclear protein that is expressed in the SGZ of the adult mouse hippocampus.

### BAF45D and GFAP are coexpressed in the adult DG

**Figure 2:**
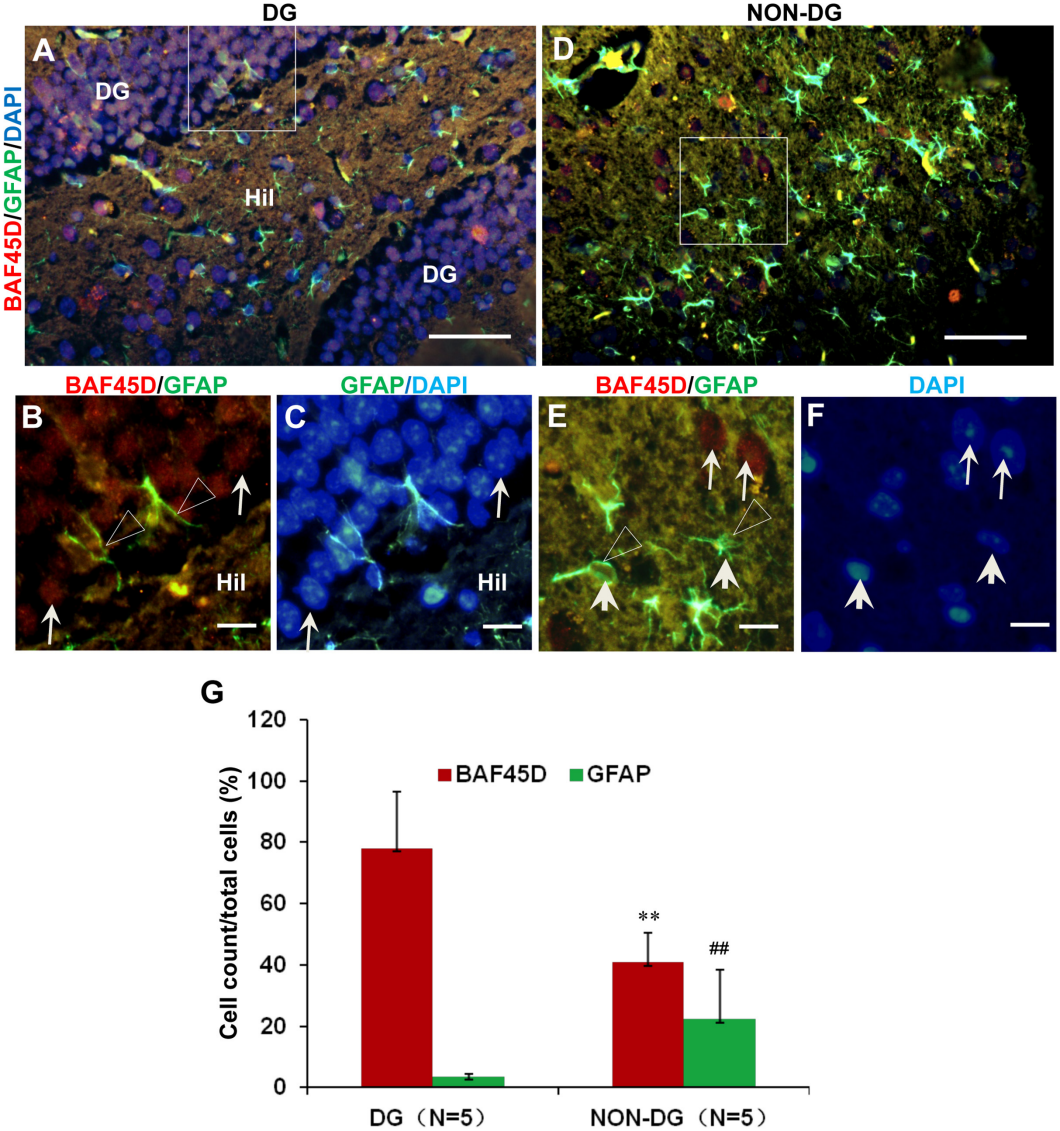
Coexpression of BAF45D and GFAP in the adult DG. **(A–C)** IF assay for the expression of BAF45D and GFAP in the DG. Panel **(B)** is a higher magnification of the merged red and green channels in the inlet in **(A)**. Panel **(C)** is a higher magnification of the merged green and blue channels in the inlet in **(A)**. The BAF45D-immunopositive signals (red) (**B**, arrows) and the GFAP-immunopositive signals (green) (**B**, triangles) were shown. **(D–F)** IF assay for the expression of BAF45D and GFAP in the non-DG regions. Panel **(E)** is a higher magnification of the merged red and green channels of the inlet in **(D)**. Panel **(F)** is a higher magnification of the blue channel of the inlet in **(D)**. The BAF45D-immunopositive signals (red) (**E**, arrows) and the GFAP-immunopositive signals (green) (**E**, triangles) were also shown. The nuclei (blue) were counterstained by DAPI **(A,C,D,F)**. Bar = 50 μm **(A,D)** and 10 μm **(B,C,E,F)**, respectively. **(G)** Quantitative assay of the ratio of the BAF45D-positive and the GFAP-positive cells in the indicated regions. Five to six mices per group were analyzed. The non-parametric statistics using Mann–Whitney *U*-test was performed using SPSS software. ^**^P < 0.01, ^##^*P* < 0.01, as compared to the DG region.

The adult NSCs in the SGZ, like radial glial cells, are characterized by the expression of GFAP (Fuentealba et al., 2012; Horgusluoglu et al., 2017). To further examine if the BAF45D-positvie cells are potential NSCs, we performed IF assay for BAF45D and GFAP using the sections of the DG and non-DG regions. The non-DG regions include lateral posterior thalamic nucleus (LP), zona incerta (ZI), and dorsal lateral geniculate nucleus (DLG). Consistent with our IH assay results, the expression of BAF45D was detected in the nuclei of most of the DG cells, while a lot of GFAP-positive cells in the Hil regions express few or no BAF45D (Figure 2A). As we expected, although most of the BAF45D-positive cells in the DG are GFAP-negative (Figure 2B, arrows), coexpression of both BAF45D (Figure 2B, arrows) and GFAP (Figure 2B, triangles) were detected in some of the SGZ cells that are next to the hilus. However, in the non-DG region (Figures 2D–F), BAF45D was found in the nuclei of some of the neural cells, which display clear nucleoli and are also devoid of GFAP expression (Figure 2E, arrows). Moreover, few or no BAF45D-positive signals were detected in the nuclei (Figures 2E,F, arrowheads) of the GFAP-positive cells (Figures 2E,F, triangles). Through the quantitative assay, while BAF45D is expressed in almost 80% of the DG cells, it is expressed only in no more than 40% of the non-DG cells (Figure 2G). On the contrary, the number of the GFAP-positive cells in the non-DG regions is much more than that in the DG region (Figure 2G).

These results suggest that BAF45D may be expressed as a nuclear protein of the GFAP-expressing NSCs in the SGZ of the adult mouse hippocampal DG.

### BAF45D is expressed in the ependymal cells of the adult brain LV

**Figure 3:**
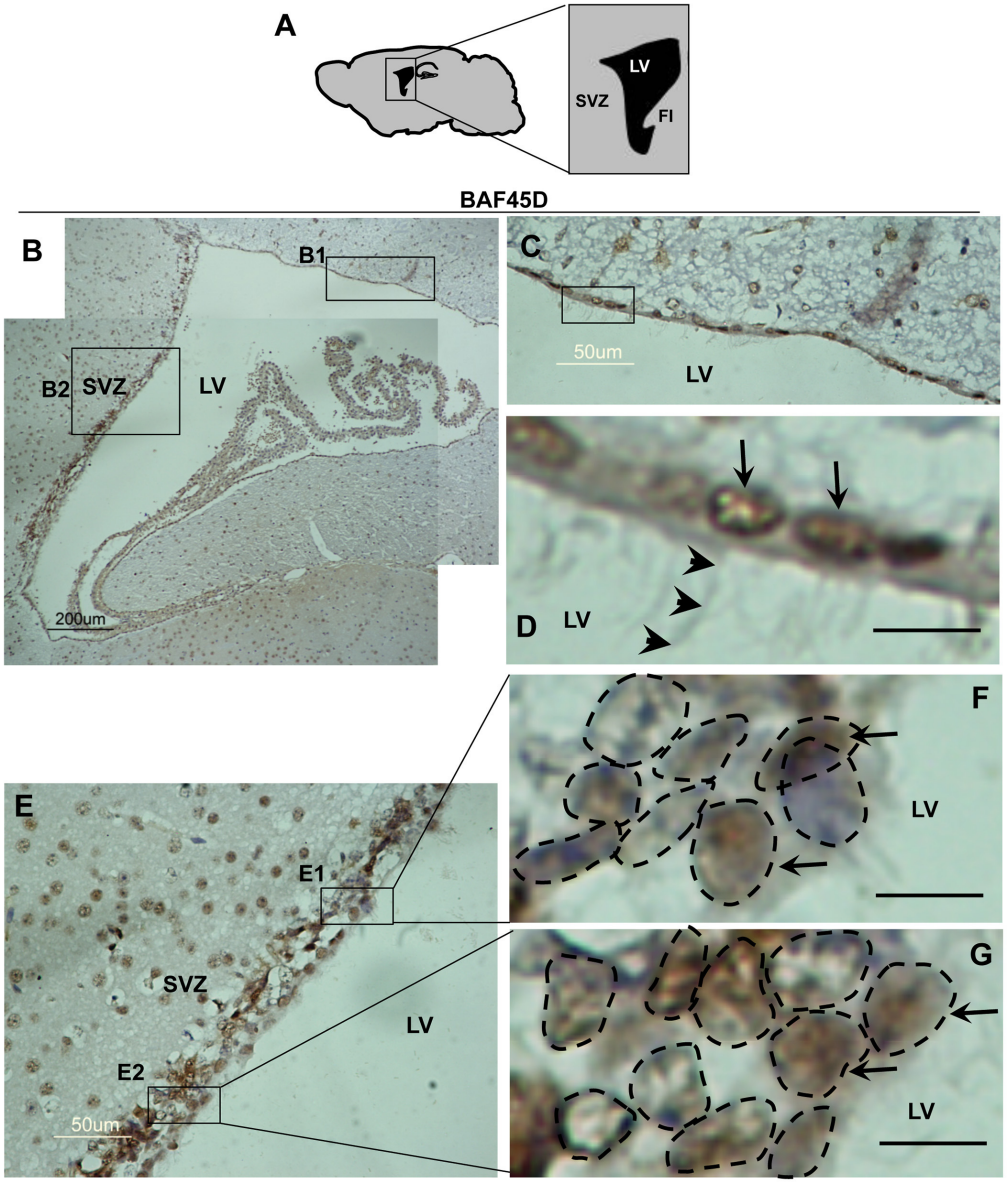
Expression of BAF45D in the ependymal cells of the adult LV. **(A)** Schematic representation of the sagittal plane (lateral 1.08 mm) of the adult mouse brain, highlighting the SVZ and LV. FI, fimbria of the hippocampus. **(B)** IH assay for examining the expression of BAF45D in the ependymal cells of the adult mouse LV. The SVZ and the LV are shown. Panel **(C)** is a higher magnification of the inlet **(B1)**. Panel **(D)** is a higher magnification of the inlet in **(C)**. The BAF45D-immunopositive signals in the nuclei of the dorsal wall ependymal cells are shown (**D**, arrows). The arrowheads indicate the cilia-like structures on the apical surface of the cells **(D)**. Panel **(E)** is a higher magnification of the inlet **(B2)**. Panel **(F)** is a higher magnification of the inlet **(E1)**. Panel **(G)** is a higher magnification of the inlet **(E2)**. The arrows indicate the BAF45D-immunopositive nuclei of the SVZ ependymal cells. The different types of the nuclei in the SVZ are shown (**F,G**, dashed circles). Bar = 10 μm **(D–G)**.

The SVZ zone along the LV is another classical adult NSC niche (Bonaguidi et al., 2016), in which the ependymal cells are essential niche components for neurogenesis under injury conditions (Carlen et al., 2009; Paez-Gonzalez et al., 2011). To examine the expression of BAF45D in the LV ependymal cells, the sagittal sections of adult mouse brain were subjected to IH assay with anti-BAF45D antibodies (Figure 3A). The results indicate that the BAF45D-immunopositive signals were detected in the ependymal cells that line both the dorsal wall and SVZ region (Figures 3B,B1,B2) of the LV, including the subependymal cells. Notably, the ependymal cells and the subependymal cells of the SVZ region form a specific nuclear architecture (Figures 3E,F,G, dashed circles), which is different from that of the dorsal wall (Figures 3C,D). At higher magnification, the BAF45D-immunopositive signals were identified mainly in the nuclei (Figures 3D,F,G, arrows). Moreover, the cilia-like structures on the apical surface of some of the ependymal cells were identified at higher magnification (Figure 3D, arrowheads).

These results suggest that BAF45D is expressed as a nuclear protein in both the ependymal cells and subependymal cells of the adult mouse LV.

### BAF45D and GFAP are coexpressed in the ependymal cells of the adult brain LV

**Figure 4:**
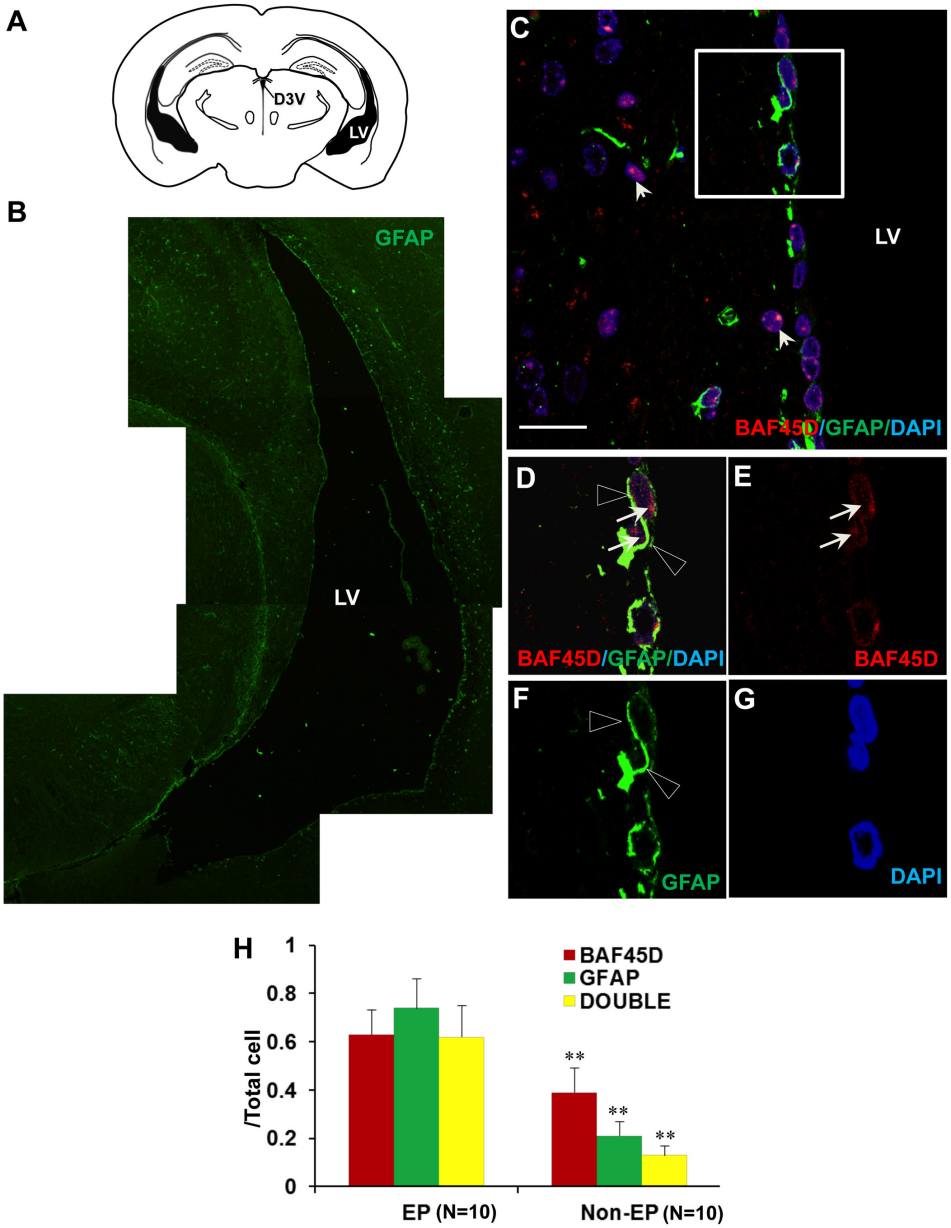
Coexpression of BAF45D and GFAP in the ependymal cells of the adult brain LV. **(A)** The schematic representation of the coronal plane (interaural 1.34 mm and bregma −2.46 mm) of the adult mouse brain, highlighting the LV and the dorsal third ventricle (D3V). **(B)** IF assay using anti-GFAP antibody for the LV. **(C–G)** The coexpression of BAF45D and GFAP in the ependymal cells of the LV. C is a subregion of the lateral wall of the LV taken by the confocal microscope. Panel **(D)** is a higher magnification of the inlet in **(C)**. **(E–G)** are the indicated different signal channels that merged in **(D)**, respectively. The BAF45D-immunopositive signals (**D,E**, arrows) and the GFAP-immunopositive signals (**D,F**, triangles) are shown. The arrowheads indicate the cells that are positive for BAF45D but negative for GFAP **(C)**. The nuclei are counterstained by DAPI. Bar = 20 μm **(C)**. **(H)** The quantitative analysis of the ratio of the BAF45D-positvie cells, the GFAP-positive cells and the double-positive cells to the total cells in the ependymal cells and the non-ependymal cells. Five to six mices per group were analyzed. Ten different areas of the lateral wall of the LVs were selected randomly for visualization. Data for comparison were implemented by independent sample *T*-test. EP, ependymal cells; NEP, non-ependymal cells. ^**^P < 0.01 as compared to the EP.

Because accumulating experimental evidences suggest that GFAP-expressing SVZ astrocytes may behave as NSCs (Bonaguidi et al., 2016), we therefore investigated the expression of both BAF45D and GFAP in the SVZ ependymal cells using the sections of the adult mouse brain cut in a coronal plane(Figure 4A). The expression of GFAP in the LV region was shown (Figure 4B). As expected, coexpression of BAF45D and GFAP in the ependymal cells was identified (Figures 4C–G). Localization of BAF45D is mainly in the nuclei (Figures 4D,E,G, arrows), while the GFAP expression is mainly in the cytoplasm and the cell processes (Figures 4D,F, triangles). This finding is similar with that of the D3V ependymal cells, which express both BAF45D and GFAP, with the BAF45D-immunopositive signals mainly found in the nuclei (Figures S2B–D, arrows) and the coexpressed GFAP-immunopositive signals found in the cytoplasm and cell processes (Figures S2C,E, triangles). The nucleus architecture of the LV is also shown (Figure 4G, dashed circles). Moreover, the nucleus architecture of the D3V, which is composed of one layer cells that are positive for both BAF45D and GFAP, seems different from that of the LV (Figure S2). Besides, there are some of the BAF45D-positive cells express no GFAP in the non-ependymal region (Figures 4D–F, arrowheads). At last, the results of the quantitative analysis indicate that among the total cells, the BAF45D-positvie cells, the GFAP-positive cells and the double-positive cells in the ependymal region are more than those in the non-ependymal region (Figure 4H).

These results suggest that BAF45D is expressed as a nuclear protein in GFAP-expressing NSCs lining the ventricular walls of the adult mouse brain.

### BAF45D is expressed in the ependymal cells of the adult spinal cord CC

**Figure 5:**
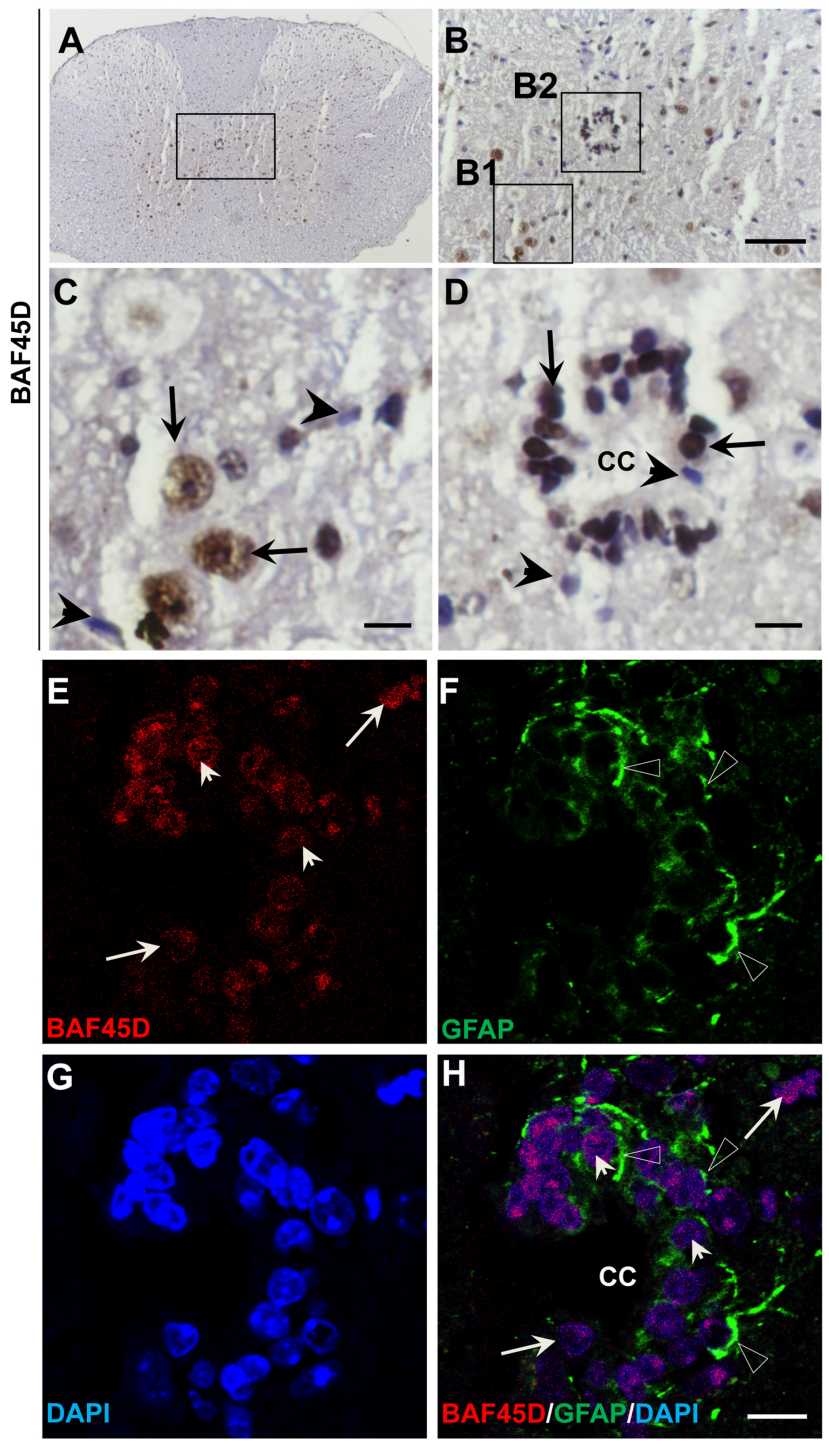
BAF45D is expressed in the ependymal cells of the adult spinal cord CC. **(A–D)** The cross sections of the adult mouse spinal cord were subjected to IH assay using anti-BAF45D antibodies. Panel **(B)** is the higher magnification of the inlet in **(A)**. **(C,D)** are higher magnifications of the inlets **(B1,B2)**, respectively. The arrows indicate the BAF45D-immunopositive nuclei of the non-ependymal cells **(C)** and the ependymal cells of the CC **(D)**. The arrowheads indicate the BAF45D-immunonegative nuclei **(C,D)**. Bar = 50 μm **(B)** and 10 μm **(C,D)**. **(E–H)**, A confocal visualization of the expression of BAF45D in the GFAP-positive cells. The triangles indicate the robust expression of GFAP in the cell processes **(F,H)**.The arrowheads indicate the BAF45D-positive cell nuclei that have the coexpressed GFAP-positive cell processes **(E,H)**. While the arrows indicate the BAF45D-positive nuclei that have few or no expression of GFAP **(E,H)**.

The central canal (CC) ependymal cells may also behave like NSCs (Fu et al., 2003; Panayiotou and Malas, 2013). To determine if BAF45D is also expressed in the CC ependymal cells, the cross sections of the adult mouse spinal cord were subjected to IH assay using anti-BAF45D antibodies (Figures 5A–D). The results proved the expression of BAF45D in the nuclei of some of the neural cells (Figure 5C, arrows) and the CC ependymal cells (Figure 5D, arrows), while there are still some of the neural cells seem BAF45D-negative (Figures 5C,D, arrowheads). Next we performed IF assay to check if the CC ependymal cells express both BAF45D and GFAP. As the data revealed, coexpression of BAF45D and GFAP was found in some of the CC ependymal cells (Figures 5E,F,H, arrowheads and triangles). While some of the GFAP-positive neural cells have few or no expression of BAF45D (Figures 5E,F,H, triangles), there are also some BAF45D-positive neural cells express no GFAP (Figures 5E–H, arrows).

These results suggest that BAF45D is expressed as a nuclear protein in putative GFAP-expressing NSCs lining the CC of the adult mouse spinal cord.

### BAF45D is preferentially expressed in the mature neurons of the adult mouse brain

**Figure 6:**
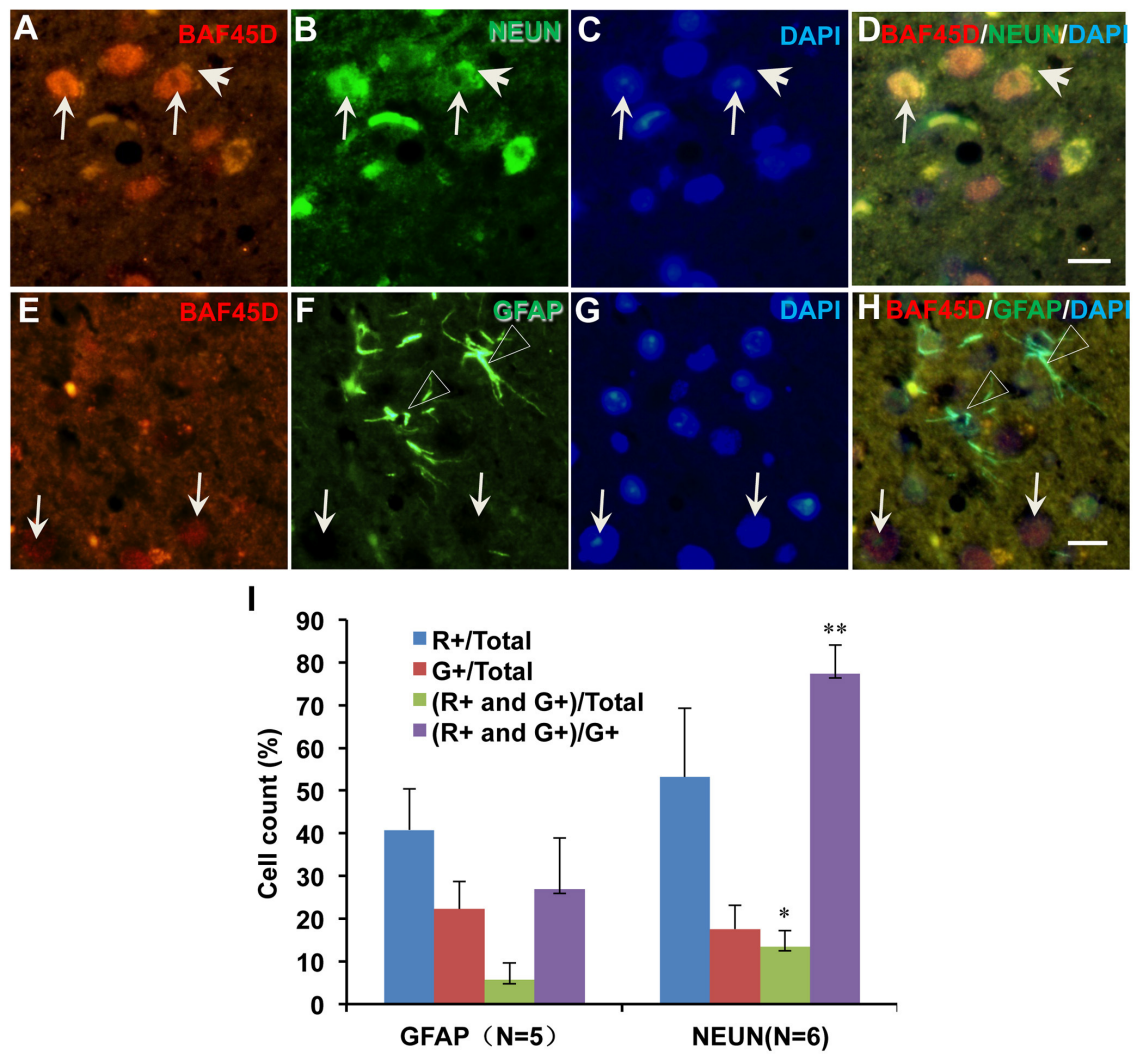
Expression of BAF45D in the neurons and astrocytes in the adult mouse brain. The brain sections are similar with the coronal section used in Figure 4. The examined brain parts include the LP (laterorostral, mediocaudal, and mediorostral parts), the ZI (dorsal and ventral parts) and the DLG. **(A–D)** Expression of BAF45D and NEUN in the neurons of the adult mouse brain. The arrows indicate the coexpression of the BAF45D and NEUN (**A,B,D**, arrows) in the nuclei which have clear nucleoli (**C**, arrows). **(E–H)** Expression of BAF45D and GFAP in the astrocytes of the adult mouse brain. The BAF45D-immunopositive nuclei show few or no GFAP-immunopositive signals (**E,G,H**, arrows). The GFAP-immunopositive cells were shown (**F,H**, triangles), which have few or no expression of BAF45D **(E,G,H)**. Bar = 10 μm. **(I)** Quantitative assay of the ratio of the cells that are positive for red channel (R+), green channel (G+), red and green channel (R+ and G+) among the total cells and the cells positive only for green channel (G+), respectively. Five to six mices per group were analyzed. The non-parametric statistics using Mann–Whitney *U*-test was performed using SPSS software. ^*^P < 0.05, ^**^P < 0.01, as compared to the GFAP.

We next want to know if BAF45D is differentially expressed between mature neurons and astrocytes. We thus performed IF assay using anti-NEUN and anti-GFAP antibodies together with anti-BAF45D antibodies. The brain sections are similar with the coronal section used in Figure 4. The examined brain parts include the LP (laterorostral, mediocaudal, and mediorostral parts), the ZI (dorsal and ventral parts) and the DLG. The data indicate that the colocalization of BAF45D and NEUN was identified in the nuclei (Figures 6A,B,D, arrows). The colocalization of cytoplasmic BAF45D and NEUN was also shown (Figures 6A,B,D, arrowheads). However, BAF45D-positive cells express few or no GFAP (Figures 6E–H, arrows), while few or no expression of BAF45D was detected in the nuclei of the GFAP-expressing astrocytes (Figures 6E,F,H, triangles). By the quantitative assay among the total cells, the ratio of the BAF45D-expressing neurons is higher than that of the BAF45D-expressing astrocytes, and the ratio of the neurons that are positive for both BAF45D and NEUN is much more than that of the astrocytes that are positive for both BAF45D and GFAP, although the ratio of the neurons has no significant differences from that of the astrocytes (Figure 6I).

These results suggest that BAF45D is preferentially expressed in the mature neurons of the adult mouse brain.

### BAF45D is preferentially expressed in the mature neurons of the adult spinal cord

**Figure 7:**
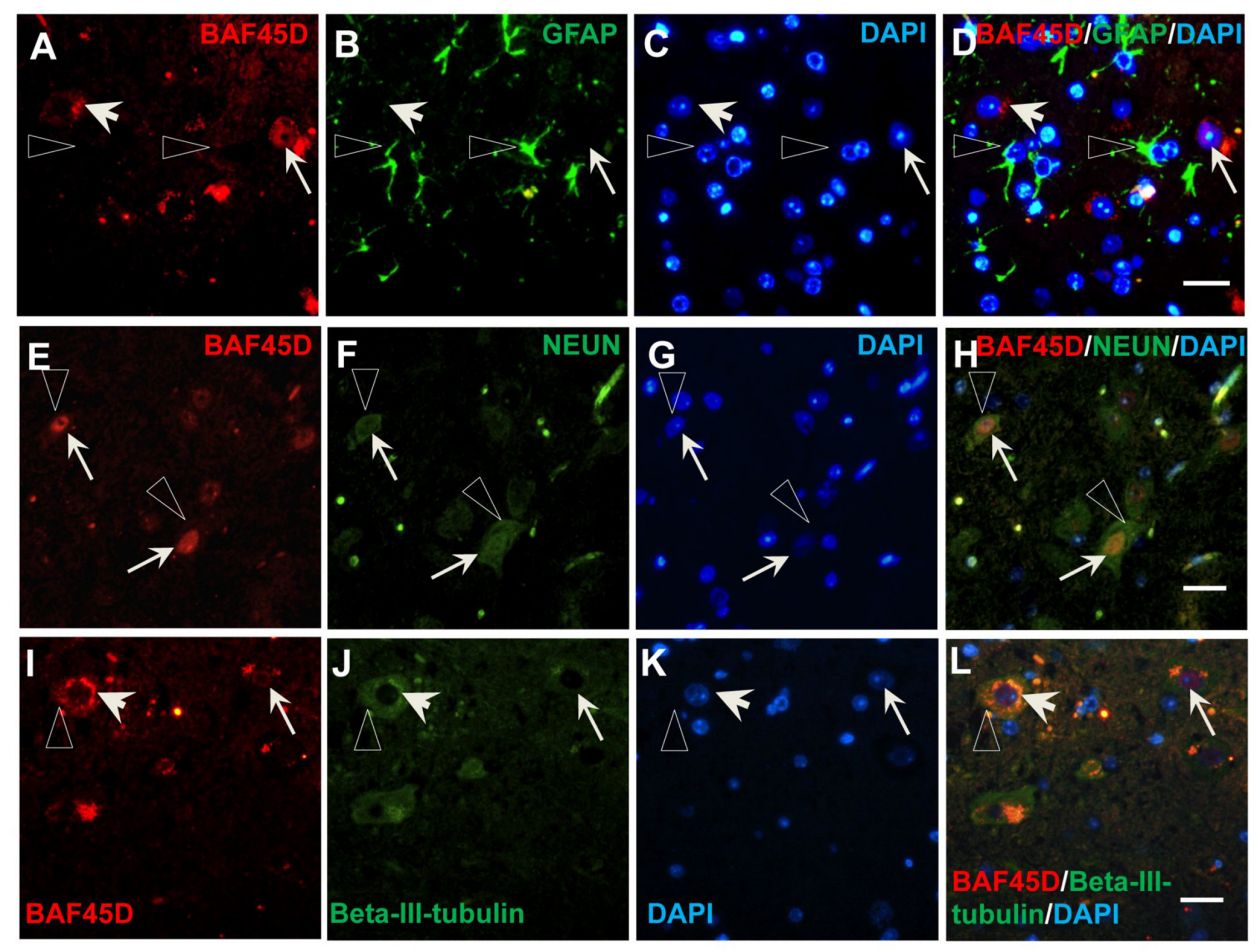
Expression of BAF45D in the neurons and astrocytes of the adult spinal cord. **(A–D)** The expression of BAF45D in the GFAP-positive astrocytes. The arrows indicate the BAF45D-positive cells **(A,C,D)**. While the arrowheads indicate the cell nuclei have few or no expression of BAF45D **(A,C,D)**, the triangles indicate the robust expression of GFAP in the cell processes **(B,D)**. **(E–H)** The expression of BAF45D in the NEUN-positive neurons. The arrows indicate the BAF45D-positive cell nuclei **(E,H)**, which are also positive for NEUN **(F,H)**. The triangles indicate the expression of NEUN in the cell cytoplasm **(B,D)**. **(I–L)** The expression of BAF45D in the beta-III-tubulin-positive neurons. The arrows indicate the BAF45D-positive cell nuclei **(I,L)**. Colocalization of beta-III-tubulin, which is expressed in the cytoplasm (triangles, **J,L**), with the cytoplasmic BAF45D was shown (**I–L**, arrowheads). Bar = 10 μm.

To prove the different BAF45D expression between neurons and astrocytes, we performed IF assay using the transverse sections of the adult mouse spinal cord. The expression of BAF45D, together with GFAP, NEUN and BETA-III-tubulin was examined. The results showed that while GFAP is expressed in both the gray matter and the white matter (Figures S3A–D), BAF45D together with beta-III-tubulin are mainly expressed in the gray matter (Figures S3E–H). Consistent with the findings in the adult brain, the BAF45D is expressed in both the nuclei and the cytoplasm of some of the cells (Figures 7A,C,D, arrows and arrowheads), while the GFAP-expressing astrocytes have few or no BAF45D expression (Figures 7B,D, triangles). Among the neurons, colocalization of BAF45D and NEUN was identified in the nuclei (Figures 7E,H, arrows), and coexpression of BAF45D and beta-III-tubulin in the neurons was also detected (Figures 7I–L, arrows and triangles). Moreover, colocalization of beta-III-tubulin with BAF45D in the cytoplasm was also shown (Figures 7I–L, arrowheads). The expression of BAF45D, GFAP and NEUN in the adult hippocampus and spinal cord has been confirmed by IB assay (Figure S4).

These results suggest that BAF45D is preferentially expressed in the neurons of the adult mouse spinal cord.

### BAF45D is required for PAX6 expression during the differentiation of H9 cells induced by RA

**Figure 8:**
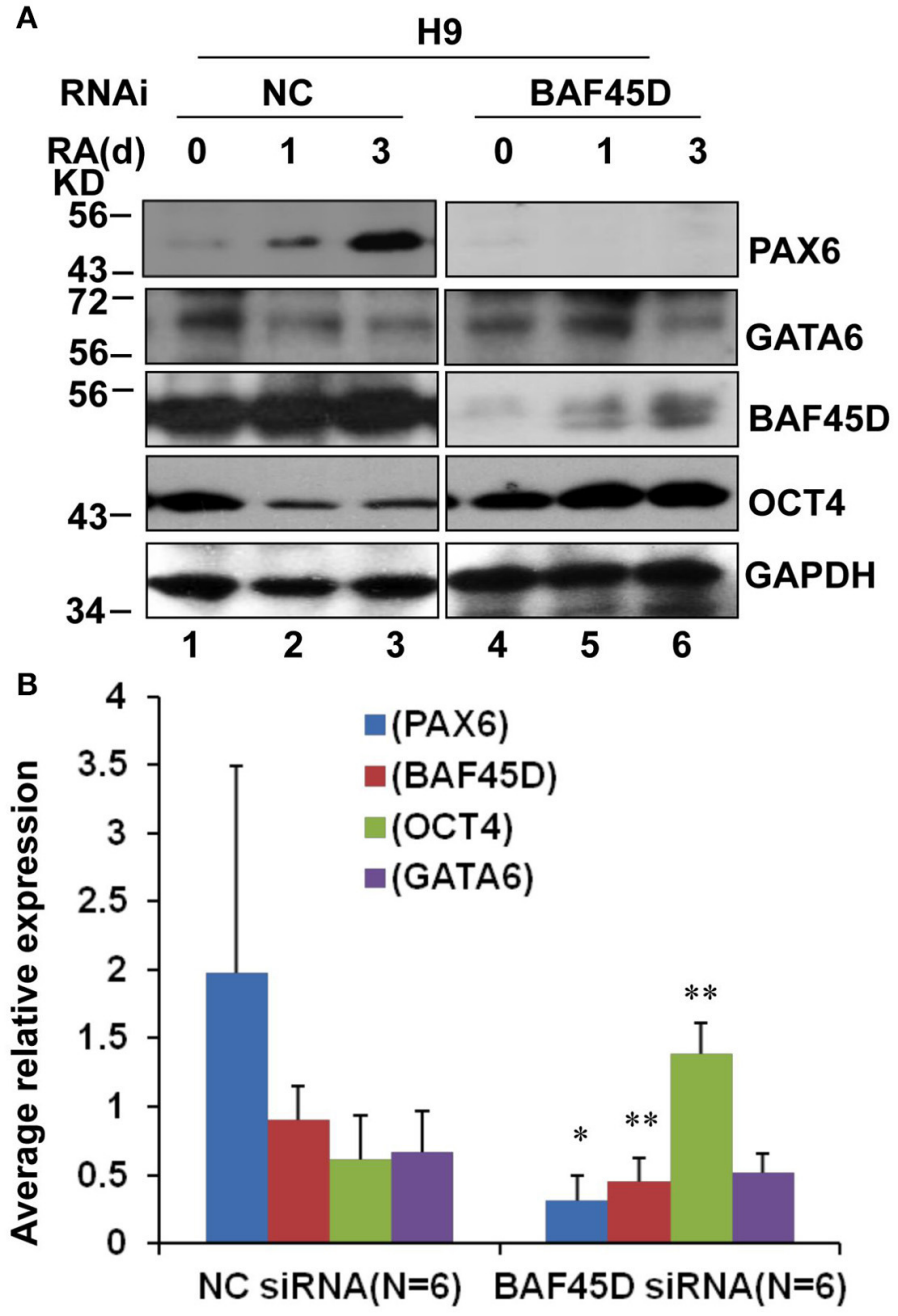
BAF45D is required for the expression of PAX6 during the RA-induced H9 cell neuroectoderm differentiation. **(A)** H9 cells infected by lentiviruses containing negative control (NC) siRNA and BAF45D #25540 siRNA were subjected to a 3-day differentiation induced by RA (1 μM). The H9-derived cells were harvested at the different time points and subjected to IB assay for the indicated proteins. **(B)** Quantitative analysis of the average expression of the indicated proteins at the different time points (d0, d1, and d3) relative to the expression at the beginning (d0) (*n* = 6). Data for comparison were implemented by one-way ANOVA. ^*^P < 0.05, ^**^P < 0.01, compared to the NC siRNA group.

We had reported that BAF45D is involved in RA-induced H9 cell general differentiation (Liu et al., 2015). Here, the expression of BAF45D in the NSC niches may imply that BAF45D can determine NSC commitment. PAX6 contributes to both embryonic and adult neurogenesis (Osumi et al., 2008). Therefore, we next performed a RA-induced differentiation of human embryonic stem cell line, H9 cells. The expression of PAX6 and BAF45D was examined along with the induction. To address if BAF45D is required for the expression of PAX6, the H9 cells were first infected by lentiviruses containing NC siRNA and BAF45D #25540 siRNA, respectively, followed by the RA-induced differentiation. Because the data of the differentiation of H9 cells have 3 time points, we thus speculated that it may be more reliable to evaluate the overall expression of the indicated proteins at 3 time points but not at a single time point. Therefore, we analyzed the average relative expression of the indicated proteins at d0, d1, and d3. As expected, RA induced upregulation of PAX6 (Figure 8A, top panel, lane 1-lane 3) and BAF45D (Figure 8A, third panel, lane 1-lane 3) together with a downregulation of OCT4 (Figure 8A, fourth panel, lane 1-lane 3) from day 1 to day 3 in the NC siRNA group. Interestingly, RA failed to upregulate PAX6 expression (Figure 8A, top panel, lane 4-lane 6) and downregulate OCT4 expression (Figure 8A, fourth panel, lane 4-lane 6) in the BAF45D siRNA group (Figure 8A, third panel, lane 4-lane 6). Quantitative analysis of the expression of the indicated proteins at the different time points relative to that of day 0 further supports the requirement of BAF45D for the PAX6 induction (Figure 8B). Upon the BAF45D knockdown, PAX6 was significantly downregulated and OCT4 was upregulated significantly at the same time. However, expression of GATA 6, an endoderm marker protein (Tiyaboonchai et al., 2017), seems not affected significantly by the BAF45D knockdown (Figure 8B).

These results suggest that RA-induced PAX6 expression in the H9 cells depends on BAF45D.

## Discussion

Here, we have characterized a new BAF45 family member, BAF45D, which is expressed in adult mouse brain NSC niches. We identified the first time the expression of BAF45D in the SGZ of the DG through both IH and IF assays (Figure 1). Moreover, coexpression of GFAP and BAF45D was also detected in the SGZ (Figure 2). Thus, we speculate that the cells that are double positive for both BAF45D and GFAP in the SGZ may be potential NSCs. It was reported that the ependymal cells forms a simple ciliated epithelium that lines the ventricular surface of the CNS, extending from the lateral ventricles to the filum terminale (Del Bigio, 2010), and also functioned as NSCs (Johansson et al., 1999). Recent progresses suggest that the ependymal cells in the SVZ may be a cell type showing NSC characterizations (Bonaguidi et al., 2016).

Next, we found that coexpression of BAF45D and GFAP in the ependymal cells of the LV and the D3V (Figure 4, Figure S2), also in the ependymal cells of the adult spinal cord CC (Figure 5). Allen Brain Atlas had been reported to be used for gene expression analysis in the adult mouse brain (Lein et al., 2007). We thus explored the online database of the Allen Brain Atlas (http://www.brain-map.org). The in situ hybridization (ISH) data of *BAF45D* gene expression in C57BL/6J adult mouse brain (http://mouse.brain-map.org/experiment/show/68301353, sagittal section) imply that *BAF45D* is preferentially expressed in the hippocampal DG. The *BAF45D*-positive cell nuclei can be identified in the SGZ region, which corroborates our data that BAF45D protein is expressed in the SGZ of the DG in the C57BL/6 adult mouse brain. Next, we also explored the ISH data of *GFAP* gene expression in the adult mouse brain (http://mouse.brain-map.org/experiment/show/79913385, sagittal section). Although, the expression pattern of *GFAP* is largely different from that of *BAF45D, GFAP* is also expressed in a few cells in the SGZ of the DG and the ependymal cells lining the LV, which is confirmed by another ISH data of *GFAP* expression (http://mouse.brain-map.org/experiment/show/79591671, coronal section). These findings are consistent with our data of the GFAP expression pattern and may also support the possibility of the coexpression of BAF45D and GFAP in the ependymal cells of the LV. Because ablation of GFAP-positive cells results in loss of NSCs in adult brain (Morshead et al., 2003) and GFAP-expressing cells can behave as NSC niche cells (Jiao and Chen, 2008). Our data here may suggest that the ependymal cells express both BAF45D and GFAP are more likely adult NSCs (Johansson et al., 1999). Adult NSCs can generate neurons, astrocytes and oligodendrocytes. Interestingly, among the GFAP-expressing cells other than the NSC-like cells, the expression of BAF45D is weak or even undetectable. On the contrary, among the neurons, BAF45D colocalizes or coexpresses with NEUN and beta-III-tubulin, two neuron marker proteins. The quantitative analysis data further indicate BAF45D is preferentially expressed in neurons in the adult mouse CNS (Figures 6, 7). Our finding that BAF45D is more highly expressed in neurons than astrocytes in non-neurogenic regions raises the possibility that the expression of BAF45D in putative adult NSCs could play a role in neuronal fate determination during adult neurogenesis, which is to be explored in our future study.

PAX6 is also a neuroectoderm marker protein (Bhinge et al., 2014; Liu et al., 2014). We therefore investigated BAF45D expression during the H9 cell neuroectoderm differentiation induced by RA. According to our data, RA induces the significant upregulation of PAX6 in the H9-derived cells (Figure 8). This is in line with previous report that RA is sufficient to induce neuroectoderm (Parsons et al., 2012). It is known that the balance between neurogenesis and NSC self-renewal depends on the PAX6 levels (Pourabdolhossein et al., 2017). According to our data, the upregulation of PAX6 levels induced by RA is dependent on BAF45D, suggesting the first time that BAF45D, as a subunit of BAF complex, is involved in RA-PAX6 signaling pathway regulating neuroectoderm development. Although some of the PAX6-positive cells can be detected until adult life, the intensity of PAX6 expression decreases with the development of the brain (Duan et al., 2013). This is consistent with our result that although weak, PAX6-positive signals are expressed in the adult hippocampal DG. PAX6 contributes to both embryonic and adult neurogenesis (Osumi et al., 2008), possibly rely on the regulation of transcription factors that required for neurogenic fate specification (Ninkovic et al., 2013; Agoston et al., 2014). In our previous study, nuclear distribution of BAF45D has been addressed in H9 cells *in vitro* (Liu et al., 2015). Here our data confirmed the nuclear distribution of BAF45D in the SGZ and SVZ cells of the adult brain and the ependymal cells of the adult spinal cord *in vivo*, which may be crucial for its transcription regulation. Besides, as a subunit of the chromatin remodeling complex, nuclear localization is also of importance for its epigenetic regulation in neurogenesis (Narayanan and Tuoc, 2014; Yao et al., 2016).

Recent report showed that the PAX6-expressing cells increased significantly as a result of acute injury in the adult human brain. Moreover, PAX6 mutations are correlated with memory dysfunction, with the most severe genotypes have the most widespread differences in a greater age-related decrease in frontoparietal cortex thickness (Yogarajah et al., 2016). In the substantia nigra (SN) from patients with Parkinson's disease (PD), the number of PAX6-positive cells was reduced compared to age and sex matched control. Furthermore, PAX6 protects SH-SY5Y cells from PD relevant neurotoxins through inhibiting apoptosis and oxidative stress (Thomas et al., 2016). Thus, our data here may imply that the BAF45D may have significant implications for contribute to PAX6 expression, which may have significant implications for treatment of neurodegenerative diseases.

Taken together, our results here may be a step forward to understand the expression of BAF45D in the adult neurogenic zones and the contribution of BAF45D to early neural development.

## Author contributions

CL: Conceived the study; CL, YS, and CS: Supervised the study; CL: Designed experiments; CL, RS, JH, DZ: Performed experiments; CL, RS, JH, DZ: Analyzed data; DH, WQ, SW, FX: Provided materials; CL: Wrote the manuscript.

### Conflict of interest statement

The authors declare that the research was conducted in the absence of any commercial or financial relationships that could be construed as a potential conflict of interest.

## Abbreviations

CNS: central nervous system
NSC: neural stem cell
SGZ: subgranular zone
DG: dentate gyrus
SVZ: sub ventricular zone
LV: lateral ventricle
GFAP: glial fibrillary acidic protein
OPC: oligodendrocyte precursor
BAF: Brg1/Brm-associated factor
ESC: embryonic stem cell
IH: immunohistochemistry
IF: immunofluorescence
IB: immunoblotting
RA: retinoid acid
D3V: dorsal third ventricle
RGL: Radial glial-like cell
SN: substantia nigra
PD, Parkinson's disease. EP: ependymal cells
NEP: non-ependymal cells.
LP: lateral posterior thalamic nucleus
ZI: zona incerta
DLG: dorsal lateral geniculate nucleus.
