# Supplementary material for: The BAF45D Protein Is Preferentially Expressed in Adult Neurogenic Zones and in Neurons and May Be Required for Retinoid Acid Induced PAX6 Expression
Source: Front Neuroanat. 2017 Nov 6;11:94. doi: 10.3389/fnana.2017.00094 (PMC5681484; doi:10.3389/fnana.2017.00094)
Supplement: Supplementary file 1 [file DataSheet1.PDF]

Supplementary

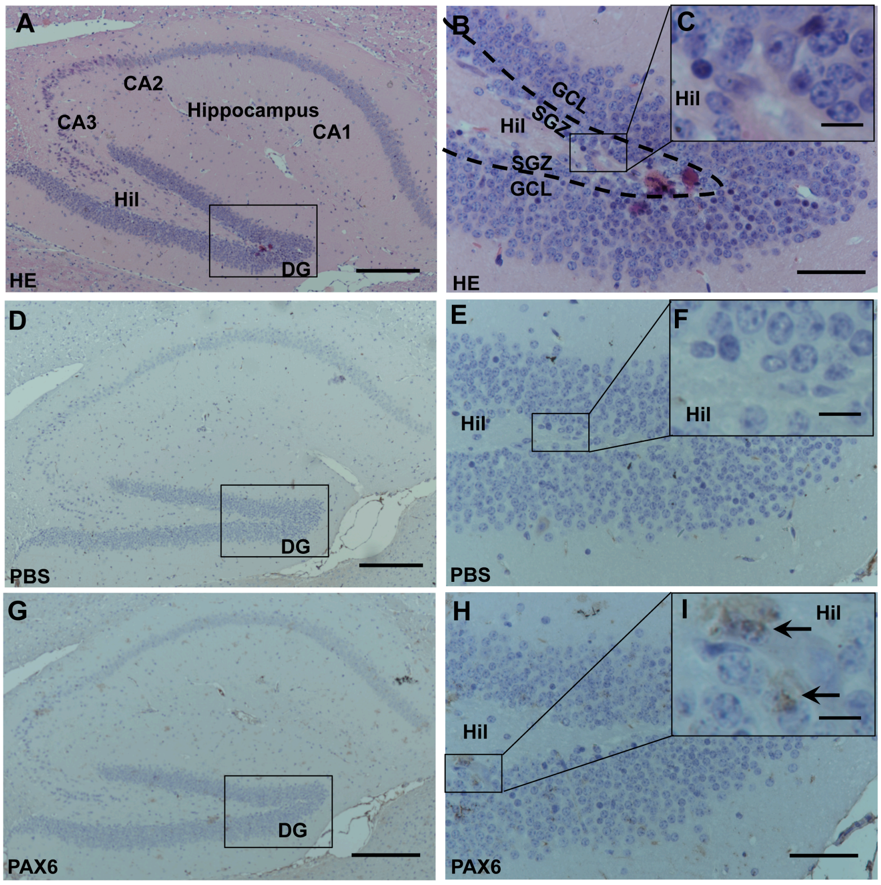

Figure S1. Expression of PAX6 in the neurons of the adult mouse hippocampus.

A-C, The sagittal sections of the adult mouse hippocampus were subjected to HE staining. The CA1, CA2, CA3 and DG of adult mouse hippocampus are shown (A). B is a higher magnification of the inlet in A. The DG is characterized by the SGZ and the GCL layer, which are isolated by a dashed line (B). C is a higher magnification of the inlets in B. The nuclear architecture of the SGZ is shown (C). ***D-I,*** The sagittal sections of the adult mouse hippocampus were subjected to IH using anti-PAX6 antibodies. Compared with the PBS control, of which the nuclear architecture is also shown (D-F), only a few of the PAX6-immunopositive signals were detected in the SGZ region (I, arrows). Bar=200 µM (A, D and G), 50 µM (B, E and H) and 10 µM(C, F and I), respectively.

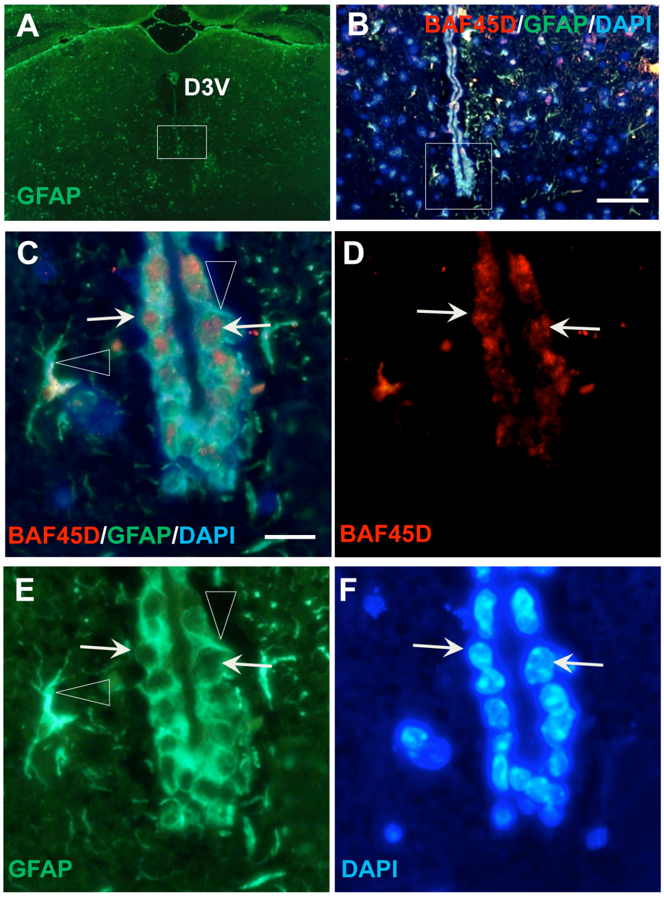

Figure S2. Coexpression of BAF45D and GFAP in the ependymal cells of the D3V.

A, IF assay using anti-GFAP antibody for the D3V. *B-F*, The coexpression of BAF45D and GFAP in the ependymal cells of the D3V. B is a higher magnification of the region that is as same as the inlet in A. C is a higher magnification of the inlet in B. D, E and F are the indicated different signal channels that merged in C, respectively. The BAF45D-immunopositive signals (C and D, arrows) and the GFAP-immunopositive signals (C and E, triangles) are shown. The nuclei are counterstained by DAPI. Bar=50 μM (B) and 10 μM (C), respectively.

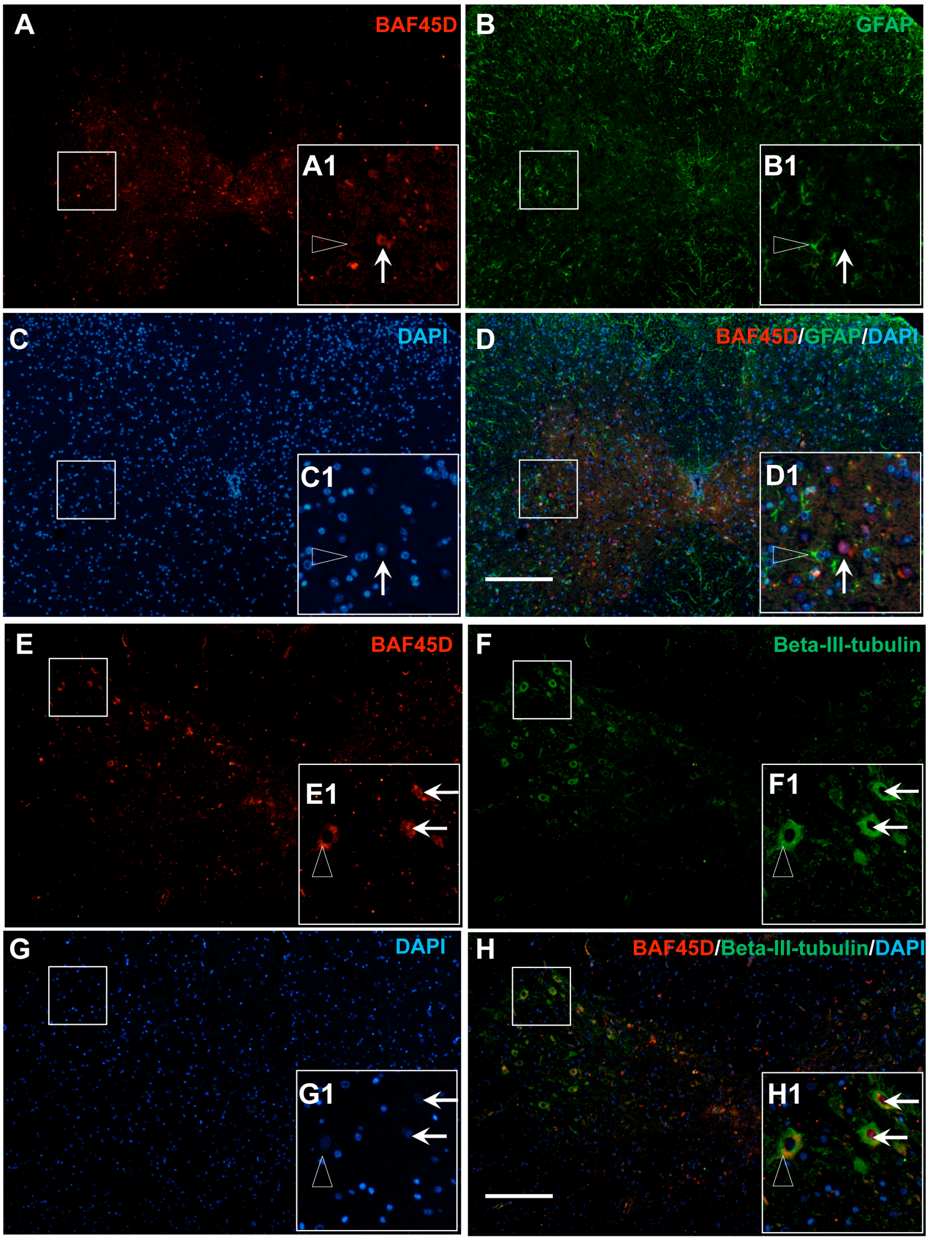

Figure.S3 Expression of BAF45D is mainly in the gray matter of the adult mouse spinal cord. ***A-D***, The transverse sections of the adult mouse spinal cord were subjected to IF assay for examining the expression of BAF45D and GFAP. A1, B1, C1 and D1 are higher magnifications of the inlets in A, B, C and D, respectively. The arrows indicate the BAF45D-positive nuclei. The triangles indicate the GFAP-positive cell processes. ***E-H***, The transverse sections of the adult mouse spinal cord were subjected to IF assay for examining the expression of BAF45D and beta-III-tubulin. E1, F1, G1 and H1 are higher magnifications of the inlets in E, F, G and H, respectively. The arrows indicate the BAF45D-positive nuclei. The triangles indicate the double positive cytoplasm. Bar=200 μM.

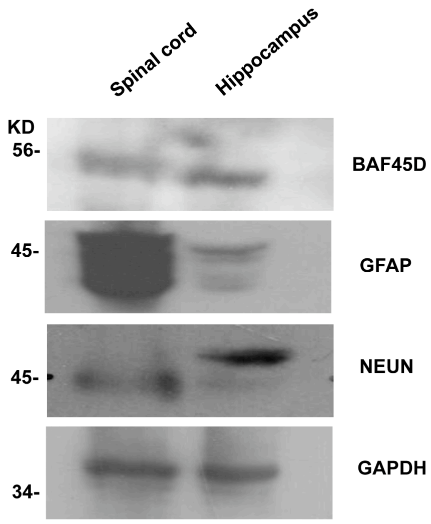

Figure. S4 IB assay for the expression of the DAF45D, GFAP and NEUN in the adult mouse spinal cord and brain.

The lysates of the fresh tissues from the adult mouse spinal cord and brain were subjected to IB assay using anti-BAF45D, anti-GFAP, anti-NEUN antibodies. The anti-GAPDH antibody was used as a loading control.

Table. S1 Description of the antibodies.

Name of the antibody	*Catalog number*	*Host*	*Species specificity*	*Immunogen*	*Publications*
BAF45D	12111-1-AP	Rabbit	Human, mouse and rat	Ag2756*	(1)
PAX6	MAB5552	Mouse	Human, rat, mouse and chick	No data	(2)
GFAP	60190-1-Ig	Mouse	Human, mouse, rat and pig	Ag10452*	(3, 4)
NEUN	MAB377	Mouse	Avian, chicken, ferret, human, mouse, pig, rat and salamander	Purified cell nuclei from mouse brain	(5, 6)
Beta-III-tubulin	MAB1637	Mouse	Avian, pig, rat, bovine, sheep, human and monkey	Amino acids 443-450 of human beta-III-tubulin	(7, 8)
Beta-III-tubulin	ARG62683	Mouse	Human, mouse, rat, pig, chick, bovine	Amino-acids 441-448 of human beta-III-tubulin	(9)
GATA6	Ab155945	Rabbit	Mouse and human	Amino acid ranges 12-31 and 561-579 (respectively) of human gata6	No data
OCT4	Sc-5279	Mouse	Human, mouse, and rat	Amino acids 1-134 of human origin	(10, 11)
GAPDH	10494-1-AP	Rabbit	Human, mouse, rat and pig	Ag0766*	(12)

*, Catalog number (Ptglab).

1. Liu C, Zhang D, Shen Y, Tao X, Liu L, Zhong Y, et al. DPF2 regulates OCT4 protein level and nuclear distribution. *Biochimica et biophysica acta* (2015) **1853**(12):3279-93. doi: 10.1016/j.bbamcr.2015.09.029. PubMed PMID: 26417682.

2. Engelkamp D, Rashbass P, Seawright A, van Heyningen V. Role of Pax6 in development of the cerebellar system. *Development* (1999) **126**(16):3585-96. Epub 1999/07/20. PubMed PMID: 10409504.

3. Yin F, Guo L, Meng CY, Liu YJ, Lu RF, Li P, et al. Transplantation of mesenchymal stem cells exerts anti-apoptotic effects in adult rats after spinal cord ischemia-reperfusion injury. *Brain Res* (2014) **1561**:1-10. Epub 2014/03/13. doi: 10.1016/j.brainres.2014.02.047
S0006-8993(14)00324-2 [pii]. PubMed PMID: 24613403.

4. Zhu Z, Liu Y, Li K, Liu J, Wang H, Sun B, et al. Protein tyrosine phosphatase receptor U (PTPRU) is required for glioma growth and motility. *Carcinogenesis* (2014) **35**(8):1901-10. Epub 2014/05/31. doi: 10.1093/carcin/bgu123
bgu123 [pii]. PubMed PMID: 24876153.

5. Nair SG, Navarre BM, Cifani C, Pickens CL, Bossert JM, Shaham Y. Role of dorsal medial prefrontal cortex dopamine D1-family receptors in relapse to high-fat food seeking induced by the anxiogenic drug yohimbine. *Neuropsychopharmacology* (2011) **36**(2):497-510. Epub 2010/10/22. doi: 10.1038/npp.2010.181
npp2010181 [pii]. PubMed PMID: 20962767; PubMed Central PMCID: PMC3005956.

6. Cai L, Bian M, Liu M, Sheng Z, Suo H, Wang Z, et al. Ethanol-induced neurodegeneration in NRSF/REST neuronal conditional knockout mice. *Neuroscience* (2011) **181**:196-205. Epub 2011/03/15. doi: 10.1016/j.neuroscience.2011.02.059

S0306-4522(11)00229-6 [pii]. PubMed PMID: 21396985.

7. Scintu F, Reali C, Pillai R, Badiali M, Sanna MA, Argiolu F, et al. Differentiation of human bone marrow stem cells into cells with a neural phenotype: diverse effects of two specific treatments. *BMC Neurosci* (2006) **7**:14. Epub 2006/02/18. doi: 1471-2202-7-14 [pii]
10.1186/1471-2202-7-14. PubMed PMID: 16483379; PubMed Central PMCID: PMC1397850.

8. Argenti B, Gallo R, Di Marcotullio L, Ferretti E, Napolitano M, Canterini S, et al. Hedgehog antagonist REN(KCTD11) regulates proliferation and apoptosis of developing granule cell progenitors. *J Neurosci* (2005) **25**(36):8338-46. Epub 2005/09/09. doi: 25/36/8338 [pii]
10.1523/JNEUROSCI.2438-05.2005. PubMed PMID: 16148242.

9. Schmidt-Edelkraut U, Daniel G, Hoffmann A, Spengler D. Zac1 regulates cell cycle arrest in neuronal progenitors via Tcf4. *Mol Cell Biol* (2014) **34**(6):1020-30. Epub 2014/01/08. doi: 10.1128/MCB.01195-13
MCB.01195-13 [pii]. PubMed PMID: 24396065; PubMed Central PMCID: PMC3958027.

10. Genovese NJ, Domeier TL, Telugu BP, Roberts RM. Enhanced Development of Skeletal Myotubes from Porcine Induced Pluripotent Stem Cells. *Sci Rep* (2017) **7**:41833. Epub 2017/02/07. doi: 10.1038/srep41833
srep41833 [pii]. PubMed PMID: 28165492; PubMed Central PMCID: PMC5292944 Animals. N.J.G., R.M.R., and B.P.V.L.T. are listed as co-inventors on a Patent Cooperation Treaty (PCT) patent application filed by the Curators of the University of Missouri through the University of Missouri-Columbia Office of Technology Management and Industry Relations.

11. Pan H, Guan D, Liu X, Li J, Wang L, Wu J, et al. SIRT6 safeguards human mesenchymal stem cells from oxidative stress by coactivating NRF2. *Cell Res* (2016) **26**(2):190-205. Epub 2016/01/16. doi: 10.1038/cr.2016.4
cr20164 [pii]. PubMed PMID: 26768768; PubMed Central PMCID: PMC4746611.

12. Wang P, Chen Z, Meng ZQ, Fan J, Luo JM, Liang W, et al. Dual role of Ski in pancreatic cancer cells: tumor-promoting versus metastasis-suppressive function. *Carcinogenesis* (2009) **30**(9):1497-506. Epub 2009/06/24. doi: 10.1093/carcin/bgp154
bgp154 [pii]. PubMed PMID: 19546161.

Table. S1 Description of the antibodies.

Name of antibody	*Catalog number*	*Source*	*Species specificity*	Immunogen	Publication
